# Books Received

**Published:** 1865-02

**Authors:** 


### BOOKS RECEIVED.

*A Treatise on Military Surgery and Hygiene.* By FRANK HASTINGS HAMILTON, M. D., *late Lieutenant-Colonel, Medical Inspector U. S. A.; Professor of Military Surgery and Hygiene, and of Fractures and Dislocations, in Bellevue Medical College; Surgeon to Bellevue Hospital; Professor of Military Surgery, &c., in Long Island College Hospital; author of "Treatise on Fractures and Dislocations," and of a "Practical Treatise on Military Surgery," illustrated with* 127 *engravings. New York:* BAILLIERE BROTHERS, 520 *Broadway,* 1865.

The simple announcement of the publication of this work will be sufficient review for most of our readers, who will immediately peruse its pages for themselves. The reputation of the distinguished author, his former works upon surgery, and his practical familiarity with military practice will afford sufficient guarantee of the value of this work, insomuch that no words of ours, could add anything to its early appreciation by the profession.

In the first Chapter, or *Introduction,* is presented a great amount of general instruction in Military Surgery, and many interesting subjects are discussed in a manner which cannot fail to interest every medical man. The differences between civil and military surgery—qualifications of military medical officers, ambulances, hospital system, character of army surgeons, their dangers, bravery, and general education, their position or rank, authority, or rather want of authority, etc., etc., are the subjects which are here introduced and which constitute a very valuable and highly entertaining portion of the work.

The second chapter, upon *Examination of Recruits,* is very full and valuable. The whole ground has been covered, and, perhaps, a more perfect, more direct and convincing statement could not be found of the conditions which should and do disqualify for active military duty. Examining surgeons will act wisely by carefully perusing this chapter.

Chapter 3d is upon the "General Hygiene of Troops," and under this heading is considered everything relating to the food, dress and care of soldiers. There are in the volume twenty-five other chapters upon the following subjects: Bivouac Accommodation of Troops in Tents, Barracks, Billets, Huts, etc.; Hospitals; Preparations for the Field; Heygienic Management of Troops upon the March; Conveyance of Sick and Wounded Soldiers; Gun-Shot Wounds; Gun-Shot Injuries of the Head; Gun-Shot Injuries of the Face and Neck; Gun-Shot Wounds of the Thorax; Punctured and Incised Wounds of the Thorax; Gun-Shot Wounds of the Abdomen; Gun-Shot Wounds in the Male Organs of Generation; Gun-Shot Fractures; Amputations; Exsections; Arrow Wounds; Traumatic Gangrene; Hospital Gangrene; Dry Gangrene; Tetanus; Scorbutus; On the Employment of Ænesthetics in Major Amputations, and other Severe Surgical Operations after Gun-Shot Injuries; Appendix. The appendix is devoted to relation of interesting and remarkable cases in detail, showing the results of the various major operations in military practice. The book, taken altogether, is a very valuable one, and constitutes our standard authority upon military surgery.

---

*The Book of Prescriptions containing* 3000 *prescriptions, collected from the practice of the most eminent Physicians and Surgeons, English, French and American; comprising also a compendious history of the Materia Medica, lists of the doses of all officinal or established preparations, and an Index of Diseases and Remedies.* By Henry Beasley, *author of "The Druggist's Receipt Book" and "The Medical Formulary." Philadelphia:* Lindsay & Blakiston, 1865.

This is a volume of the Prescriptions of those "medical authors who are generally considered of most authority," with a short description of each medicine, and a list of the doses in which its several preparations may be prescribed. It also contains an index of Diseases and Remedies, and to this edition have been added one hundred new prescriptions.

The art of prescribing medicines scientifically and tastily, should most certainly be cultivated, and the formulæ contained in this book, are generally elegant, and every way satisfactory. It however, must not be omitted, in connection with the appearance of such work, to say that physicians should not study these forms and administer these medicines, because the most distinguished physicians have done so, and recommended them to others. We have no doubt that many of the prescriptions herein contained, may have appeared to the authors as eminently useful in the cases for which they were prescribed, and that the authority is good for their administration, at the same time, we presume that no other physician will ever observe half so favorable results. That physician, who uses the fewest remedies, and understands fully the uses of these few medicines, will prove himself the most safe and successful practitioner, while he who copies the prescriptions of the "most distinguished physicians" will make a dunce of himself, and often a victim of his patients.

This book of prescriptions is presented to us in the best style of the publishers, and is in every way an attractive volume. Though we have no great veneration for the prescriptions of distinguished authors, and do not recommend physicians to imitate or copy them, advocating at the same time the use of only a few well understood, well tested remedies; still we do not find any fault with this book. It is just what it claims to be; is in good style, the selections are well and carefully made, and the volume is thoroughly adapted to the objects intended by its publication.

---

*Glaucoma; its Symptoms, Diagnosis, and Treatment, by* PETER DIRCK KEYSER, M. M. *Philadelphia:* LINDSAY & BLACKISTON, 1864.

The author says, that in the great majority of cases there is a premonitory stage in this disease, which is characterized by several or all the following symptoms, which are, however, of periodic occurrence, there being an interval of perfect intermission. When theré are not perfect intermissions, only remissions of the symptons, it must be regarded as a confirmed glaucoma.

The following symptoms are met with in the premonitory stage:

1. Increased tension of the eyeball.
2. Marked increase of any existing presbyopia.
3. Venous hyperæmia.
4. Haziness of the aqueous and vitreous humors.
5. Dilatation and sluggishness of the pupil.
6. Periodic dimness of sight.
7. The appearance of a halo or rainbow round a candle.
8. Intermitting pains in and around the eye; these are not always present.
9. Slight contractions of the field of vision.

This dissertation or monograph is well worthy attentive perusal by all active practitioners. It is very instructive, and will greatly aid in the early diagnosis of this disease.

---

